# Clinical Profiles, Occurrence, and Management of Adolescent Patients with HAIR-AN Syndrome

**DOI:** 10.1100/tsw.2004.106

**Published:** 2004-07-08

**Authors:** Hatim A. Omar, Stephanie Logsdon, Jessica Richards

**Affiliations:** Section of Adolescent Medicine, Department of Pediatrics, University of Kentucky, Lexington, KY 40536-0284, USA

**Keywords:** adolescent medicine, hyperandrogenism, insulin resistance, acanthosis nigricans, metformin, HAIR-AN syndrome, polycystic ovary syndrome, United States

## Abstract

The syndrome of hyperandrogenism, insulin resistance, and acanthosis nigricans (HAIR-AN) is a subphenotype of the polycystic ovary syndrome. It is one of the most common causes of menstrual problems, hyperandrogenic symptoms, and insulin resistance among young women. Review of clinical data in an outpatient adolescent clinic showed that of the 1,002 young women (ages 10—21 years) attending the clinic over a 2-year period, 50 (5%) were diagnosed with HAIR-AN syndrome. Mean age of the patients was 15.5, initial mean weight at diagnosis was 94.5 kg, and the mean BMI was 33.33 kg/m. Patients were treated with a weight-stabilization and -reduction program, oral contraceptive pills, and in most cases metformin. Of the patients, 80% were compliant with the follow-up and treatment regimen, 60% maintained or reduced their weight, 95% had regular menstrual cycles, and in most patients, the acne and/or hirsutism were the same or better than at the start of treatment. We conclude that HAIR-AN syndrome is a common disease in young women and multifaceted, aggressive treatment appears to be effective in reducing the severity of symptoms and preventing further consequences.

## INTRODUCTION

The syndrome of hyperandrogenism, insulin resistance, and acanthosis nigricans (HAIR-AN) has been known for almost 30 years[1,2,3,4]. The primary abnormality in these patients is thought to be insulin resistance with elevated insulin levels[1,2]. This syndrome is a unique subphenotype of the polycystic ovary syndrome and is seen in almost 5% of all females with hyperandrogenism[2,5]. Obesity, acne, hirsutism, and acanthosis nigricans usually appear on the afflicted adolescent around puberty[6].

Hirsutism is defined as the growth of pigmented hair on the chest, back, and lower abdomen. Acanthosis nigricans describes darkly pigmented, velvety areas of the skin. This manifestation of the syndrome is associated with insulin resistance and decreased insulin sensitivity[7]. It is still not completely known how insulin resistance occurs, however, defects and genetic receptors are thought to be responsible in most patients[1,3,4]. When insulin levels are markedly elevated over time, they tend to directly stimulate the overproduction of androgens in the ovaries[3,8]. It is thought that high insulin levels cross-react with receptors for insulin-like growth factors leading to increased ovarian androgen production[2,3,8]. Approximately 5% of women with hyperandrogenism and insulin resistance also tend to have acanthosis nigricans[9,10]. Patients with HAIR-AN syndrome may present with amenorrhea and signs of verilization[2,4]. They may also have increased levels of insulin, testosterone, and androstenedione levels with normal andrenal function[3]. Adolescents with HAIR-AN syndrome usually present with normal levels of luteinizing hormone (LH) and follicle-stimulating hormone (FHS)[3], although the ratio of LH to FHS is usually more than one. There are no specific protocols for treatment of this syndrome. Current management usually involves oral contraceptive pills with or without an antiandrogen. Oral contraceptives may suppress the hypothalamic-pituitary-ovarian axis which would lower ovarian steroid production[3].

Metformin is an insulin-sensitizing drug that has also been used in the treatment of HAIR-AN syndrome. Few studies test its use in adolescents, but in small trials it appears to reduce weight gain and regulate menstrual cycles. The effect of metformin on hirsutism has not been adequately studied[11]. The reduction of insulin resistance by metformin could reduce the appearance of acanthosis nigricans on the adolescent[12]. Previous studies have shown that metformin reduces the body mass index (BMI) of HAIR-AN patients and also helps regulate the menstrual cycle[13]. Use of cosmetic methods such as laser hair removal can also be utilized to reduce the severity of existing hirsutism[11]. Weight loss is also an effective way to reduce the manifestations of HAIR-AN syndrome and also reduce the risk for other diseases including diabetes and heart disease. A lower-calorie diet rich in fiber and protein can aid in healthy weight loss for the obese adolescent[6]. It is also recommended that physicians council the adolescent about the importance of exercise and assist in the formation of an exercise program tailored to the individual patient.

Beyond physical treatment of HAIR-AN syndrome, physicians should also be observant of the mental state of the patient. Most adolescent females are concerned with their body image and are sensitive to the phenotypic manifestations of the syndrome[11]. A short evaluation of the patient’s concerns and emotions associated with the diagnosis of HAIR-AN syndrome can alert physicians to any problems. Reassurance and thorough explanations of the treatment can comfort and encourage the patient.

In this study, we reviewed clinical data from patients attending the Adolescent Medicine Clinic at the University Based Health Center to investigate the occurrence.

## METHODS

Retrospective review of medical records attending the Adolescent Medicine Clinic at the University Based Health Center in a medium-sized city was performed to assess the occurrence, clinical profiles, and management issues of patients with HAIR-AN syndrome. The study was approved with an exemption protocol from the Institutional Review Board at the University of Kentucky, Lexington.

## RESULTS

In the period between January 2000 and December 2002, a total of 1,002 young women between the ages of 10 and 21 years attended the Adolescent Medicine Clinic at the University Based Health Center. This number included all visits regardless of the reason for attending the clinic, including health maintenance, acute care, or family planning. A total of 136 patients (13.6%) presented with complaints of irregular menstrual periods; 50 patients (5% of the total and 36.8% of those with irregular periods) were diagnosed with HAIR-IN syndrome. Patients were diagnosed with HAIR-AN syndrome if they had clinical characteristics indicating hyperandrogenism, insulin resistance, and acanthosis nigricans[14].

Demographic characteristics of the patients are shown in Table 1. All 50 patients had laboratory evidence of insulin resistance (elevated insulin level) and most patients had one or more laboratory values indicative of hyperandrogenism[1,2,3,4,5,6,7,14]; 74% of the patients had at least one comorbidity (Tables 1 and 2).

**TABLE 1**
**Demographic and Clinical Profiles of Patients**

| | |
|---|---|
| Age (Mean $\pm$ SD} | 15.5 ± 2.7 Years, 10–19 |
| Race | 20 (40%) W, 27 (54%) B, 3 (6%) Other |
| Menarche | 10.8 ± 1.3 Years of age |
| Initial weight | 94.5 ± kg (208.9 ± 44.6 lb) |
| Initial BMI | 33.1 ± 4.1 kg/m$^2$ |
| Previous pregnancy | 3 (6%) Yes, 47 (94%) No |
| Current sexual activity | 15 (30%) Yes, 35 (70%) No |
| Comorbidity | 37 (74%) Yes, 13 (26%) No |
| Insurance | 28 (56%) MC, 18 (36%) Private, 4(8%) None |

**Age range given as 10-21 elsewhere.**

**TABLE 2**
**Most Common Comorbidity**

| | |
|---|---|
| Depression | 12 (24%) |
| Bipolar disorder | 2 (4%) |
| Learning disability | 1 (2%) |
| Recurrent headaches | 17 (34%) |
| Allergies | 22 (44%) |
| Hypertension | 4 (8%) |
| GERD | 4 (8%) |
| Diabetes (Type II) | 8 (16%) |

Once the diagnosis was made, patients were treated by a team consisting of an adolescent medicine physician, nutritionist, social worker, and psychologist. Treatment included nutrition changes and a physical activity regimen with the goal of maintaining, then reducing, body weight. Patients were also started on periodic psychology counseling to improve self-esteem and to address any mental health issues. All patients were started on monophasic, low androgenic, oral contraceptive pills, usually norgestimate/ethinyl estradiol or drospirenone/ethinyl estradiol. Forty patients (80%) who had fasting glucose to insulin ratio of less than five were also started on metformin (Bristol-Myers Squibb, Princeton, NJ) or generic equivalent. Patients were usually started on a dose of 500 mg once a day for 1 week, then the dose was gradually increased to a maximum of 100 mg twice a day depending on tolerance of the patient to the medication. All patients had a metabolic screen and liver function test performed prior to starting metformin and every 6 months thereafter.

Evaluation of patients every 3 months showed that 80% of the patients were compliant with all aspects of treatment throughout the follow-up period and 20% of the patients had no compliance issues either with the physical activity/nutrition plan or with taking their oral contraceptive pills. Interestingly, all patients were compliant with the psychological counseling for the allotted 6 months of treatment. All patients that were compliant with oral contraceptive pills had regular menstrual periods following treatment and their acne and/or hirsutism improved or were unchanged after treatment, 20% of the patients had improvement in their acanthosis nigricans, while the others were unchanged. Of the patients on metformin, 40% reported nausea, but no other side effects were reported and none had a problem with liver function at follow-up. Only 1 patient out of the 40 (2.5%) using metformin discontinued use because of nausea.

## DISCUSSION

Most adolescent females are usually concerned about their body image and are very sensitive to any changes that may interfere with their appearance[11]. The symptoms of HAIR-AN syndrome do exactly that, by increasing the severity of acne, by inducing hirsutism and the appearance of acanthosis nigricans, in addition to obesity[2,3,4,5,6]. In addition to the appearance issues there are also significant medical consequences such as irregular periods, infertility, and type II diabetes[2,3,4,5,6]. Early detection, diagnosis, and treatment of HAIR-AN syndrome may reduce the symptoms and improve the quality of life of these young women. Based on the results of this study, HAIR-AN syndrome appears to be a fairly common disorder in adolescents with 5% of the clinic female adolescent population suffering from the disease. This number is somewhat higher than what is reported in other studies[9,10]. In part, this may be due to the fact that our clinic is a tertiary care center and attended by patients with more morbidity referred by their primary care providers. Nevertheless, this syndrome constitutes significant problems for young women.

In this study, 30% of the patients with HAIR-AN syndrome also had a mental health comorbidity, most frequently depression, in 24% of the patients (Table 2); 70% of the patients in this study were not sexually active, which is different than their peer groups in our area, where 68% of the 16 year olds have had at least one sexual experience (unpublished observation). In general, adolescents are notoriously noncompliant with long-term treatment. In this study, however, 80% of the patients were compliant with treatment, which may be due to the multidisciplinary approach to the patient in our clinic in addition to the desire to improve their appearance and self-esteem.

The finding that metformin was well tolerated in this patient population may help future long-term prospective studies on the subject. In most patients, there was significant success of treatment both in terms of appearance and actual improvement in regulating their menstrual periods, improving their BMI, and preventing the development of type II diabetes and other severe medical consequences.

Our study is limited by the retrospective nature and the relatively small number of participants.

## CONCLUSION

HAIR-AN syndrome is a common multisystem disorder in the adolescent age group, which is associated with significant morbidity and self-esteem issues. Early detection, diagnosis, and treatment can help reduce further morbidity, improve self-esteem, and have a positive impact on the quality of life of these patients.

## BIOSKETCHES

**Hatim A Omar, MD**, Professor of Pediatrics, Obstetrics and Gynecology and Director of the Section of Adolescent Medicine, Department of Pediatrics, University of Kentucky, Lexington. Dr. Omar has completed residency training in Obstetrics and Gynecology as well as Pediatrics. He has also completed fellowships in Vascular Physiology and Adolescent Medicine. He is the recipient of the Commonwealth of Kentucky Governor's Award for Community Service and Volunteerism and is well known internationally as an authority in Adolescent Medicine and Pediatric and Adolescent Gynecology. E-mail: haomar2@uky.edu

**Stephanie Logsdon**, is an undergraduate student at the University of Kentucky, majoring in biology. She has worked during her summer research project with the Section of Adolescent Medicine, Department of Pediatrics, University of Kentucky, Lexington.

**Jessica Richards**, **RN**, Section of Adolescent Medicine, Department of Pediatrics, University of Kentucky, Lexington. She is very active in research concerning adolescents and teen mothers.
